# Single‐cell RNA sequencing analysis to characterize cells and gene expression landscapes in atrial septal defect

**DOI:** 10.1111/jcmm.16914

**Published:** 2021-09-12

**Authors:** Zunzhe Wang, Huating Wang, Ya Zhang, Fangpu Yu, Liwen Yu, Cheng Zhang

**Affiliations:** ^1^ The Key Laboratory of Cardiovascular Remodeling and Function Research Chinese Ministry of Education Chinese National Health Commission and Chinese Academy of Medical Sciences State and Shandong Province Joint Key Laboratory of Translational Cardiovascular Medicine Department of Cardiology Qilu Hospital Cheeloo College of Medicine Shandong University Jinan 250012 China; ^2^ Department of Cardiology Jinan Central Hospital Affiliated to Shandong University Jinan China

**Keywords:** atrial septal defect, cardiomyocyte, pseudotime trajectory, single‐cell RNA sequencing

## Abstract

This study aimed to characterize the cells and gene expression landscape in atrial septal defect (ASD). We performed single‐cell RNA sequencing of cells derived from cardiac tissue of an ASD patient. Unsupervised clustering analysis was performed to identify different cell populations, followed by the investigation of the cellular crosstalk by analysing ligand‐receptor interactions across cell types. Finally, differences between ASD and normal samples for all cell types were further investigated. An expression matrix of 18,411 genes in 6487 cells was obtained and used in this analysis. Five cell types, including cardiomyocytes, endothelial cells, smooth muscle cells, fibroblasts and macrophages were identified. ASD showed a decreased proportion of cardiomyocytes and an increased proportion of fibroblasts. There was more cellular crosstalk among cardiomyocytes, fibroblasts and macrophages, especially between fibroblast and macrophage. For all cell types, the majority of the DEGs were downregulated in ASD samples. For cardiomyocytes, there were 199 DEGs (42 upregulated and 157 downregulated) between ASD and normal samples. PPI analysis showed that cardiomyocyte marker gene FABP4 interacted with FOS, while FOS showed interaction with NPPA. Cell trajectory analysis showed that FABP4, FOS, and NPPA showed different expression changes along the pseudotime trajectory. Our results showed that single‐cell RNA sequencing provides a powerful tool to study DEG profiles in the cell subpopulations of interest at the single‐cell level. These findings enhance the understanding of the underlying mechanisms of ASD at both the cellular and molecular level and highlight potential targets for the treatment of ASD.

## 1 INTRODUCTION

Atrial septal defect (ASD) is one of the most common forms of congenital heart disease (CHD) and a major cause of childhood morbidity and mortality, with an estimated incidence of 100 per 100,000 live births.^1^ The process of cardiomyogenesis is precisely and spatially regulated by signalling molecules. The regulation of this process involves a conservative network of tissue‐specific transcriptional factors that are necessary for the morphogenesis of the atrioventricular septum. Any interruptions to this process can result in embryonic lethality or heart defects.^2^, ^3^ ASD is a non‐cyanotic CHD triggered by aberrant abnormal blood flow between the left and right atria.^4^ However, the pathogenic mechanism of ASD remains largely unknown, despite that great efforts have been employed in the prevention, diagnosis, and treatment of ASD.^5^, ^6^, ^7^

Studies have investigated the pathogenesis and crucial molecular markers of ASD. For example, multiple transcription factors, including GATA4, NKX2‐5, dHAND, TFAP2 and TBX5, are required for early heart development.^8^, ^9^ Yang et al. suggested that selective expression of NEXN, an F‐actin binding protein, could lead to ASD by inhibiting GATA4.^10^ Duong et al. showed that Nr2f1a is expressed in differentiated atrial cardiomyocytes and that it mediates the size of the atrial and atrial‐atrioventricular canal by regulating the differentiation of atrial cardiomyocytes.^11^

Single‐cell RNA sequencing (scRNA‐seq) analysis allows the characterization of gene expression landscapes at the single‐cell level, which can help us to understand the potential regulatory and driving mechanisms of biological disorders.^12^ Utilizing scRNA‐seq, studies have investigated the spatial and temporal programming of heart development in animal models, which has revealed the gene expression patterns in the process of organ development.^13^, ^14^ On the basis of scRNA‐seq analyses of healthy and diseased heart, Gladka et al. suggested that there were disease‐specific cell subpopulations, and found that CKAP4 could modulate the activation of fibroblasts, showing positive correlations with known myofibroblast markers.^15^ However, to our knowledge, no studies have yet investigated the development of ASD using scRNA‐seq analysis. In addition, scRNA‐seq studies of heart development have mostly been based on animal models, and less so on human cardiac tissue. Therefore, we aimed to characterize gene expression in cells derived from ASD and normal control tissues using scRNA‐seq analysis.

## 2 MATERIALS AND METHODS

### 2.1 Patient and tissue samples

Normal ventricular muscle tissue and tissues adjacent to ASD were collected from the cadaver of a 3‐month‐old male ASD patient. The study protocol was reviewed and approved by the Medical Institutional Ethics Committee of Qilu Hospital, Shandong University, China. (Prot. KYLL‐2018‐080). The study was carried out in accordance with the approved guidelines. Written informed consent was provided by the parents. All procedures in this study were performed in compliance with the Helsinki Declaration.

### 2.2 Single‐cell sequencing and data pre‐processing

Samples were prepared into a single‐cell suspension and examined for cell count and cell viability using a Countess^®^ II Automated Cell Counter. Single‐cell suspensions with cell activity above 80% and a cell concentration of 1000 cells/μl were mixed with 10× Barcode Gel Beads and enzyme to construct a 10× Genomics labelled single‐cell library in accordance with the manufacturer's instructions. The Illumina HiSeq platform was used for sequencing of the library. Raw reads were aligned to the reference genome using STAR cell ranger, and unique alignment sequences were selected for subsequent analysis. The Unique Molecular Identifier (UMI) was calibrated based on the unique RNA sequence alignment results. After removal of duplicates, UMI counting was carried out for the different genes for each Barcode to determine the effective cells.

### 2.3 Unsupervised clustering and cell‐type annotation

Expression data were normalized based on UMI, followed by the analysis of reduced dimension using principal component analysis. A graph‐based clustering algorithm^16^ and K‐means^17^ were used for cell clustering analysis. The t‐distributed stochastic neighbour embedding (tSNE)^18^ was used to visualize the clustering results. Based on the results of cell clustering, exact tests of the negative binomial of sSeq were used to perform differential analysis and identify the significantly differentially expressed genes of each cell cluster. These genes were considered feature genes. Cell clusters with more than 1% proportion of cells were selected for subsequent analysis. On the basis of edger analysis^19^ (Version: 3.4, http://www.bioconductor.org/packages/release/bioc/html/edgeR.html), the count matrices of gene expression were converted into logCPM for subsequent analysis. A total of 24 cell markers were obtained based on the feature genes combined with the cell markers recorded in the CellMarker^20^ (http://bio‐bigdata.hrbmu.edu.cn/CellMarker/) and PanglaoDB^21^ (https://panglaodb.se/) databases, and the cell markers reported in the study of Cui et al.^22^ FABP4, CD36, TNNT3 and AQP1 were markers for cardiomyocytes; SELE, ACKR1, PLVAP, DNASE1L3 and CCL14 were markers for endothelial cells; RGS5, GJA4, TAGLN, ACTA2, MYL9 and SOD3 were markers for smooth muscle cells; DCN, COL1A2, LUM, COL1A1, FBLN1 and TCF21 were markers for fibroblasts; and AIF1, CD163 and CD68 were markers for macrophages. The R package ComplexHeatmap was used to visualize heatmaps for cell marker expression, and cell clusters were annotated for significantly highly expressed markers.

### 2.4 Cell‐cell crosstalk between cell types

In order to explore the cell‐cell crosstalk among different cell types, the R package iTALK^23^ (https://github.com/Coolgenome/iTALK) was used to analyse ligand‐receptor interactions. In brief, the upregulated genes of each cell type were matched to the 2,648 non‐redundant ligand‐receptor interactions (including growth factors, cytokines, checkpoints and another four types) recorded in the iTALK package.

### 2.5 Cell trajectory analysis

The single‐cell trajectory analysis method allows the ordering of cells along with a pseudotime axis, which helps to characterize transitional processes such as lineage development.^24^ The R package Monocle^25^ (version: 2.18.0, http://bioconductor.org/packages/release/bioc/html/monocle.html) was used to perform cell pseudotime trajectory analysis. Genes that were expressed in at least ten cells with mean expression values >0.5 and differentially expressed with *q* values <0.01 were used in cell trajectory analysis.

### 2.6 Differential expression analysis between ASD and normal tissue for each cell type

Differential expression analysis between ASD and normal tissue for each cell type was performed using the R package edgeR. The Benjamini and Hochberg method was used to perform multiple tests correction. Differentially expressed genes (DEGs) were selected that had |logFC| >0.263 (1.2 fold change) and adjusted *p*‐values <0.05.

### 2.7 Functional enrichment analysis

Gene Ontology (GO_BP) terms and KEGG pathways were analysed for DEGs identified in each cell type using the online Metascape tool^26^ (http://metascape.org) with default parameters: Min Overlap = 3, *p*‐value Cut‐off = 0.05, and Min Enrichment = 1.5. The top 10 GO_BP terms and KEGG pathways (ranked by *p*‐value) were displayed in bubble diagram.

### 2.8 Gene set variation analysis (GSVA)

The R package GSVA^27^ (version: 1.36.2, http://bioconductor.org/packages/release/bioc/html/GSVA.html) was used to perform gene set variation analysis (GSVA) to compare GO_BP terms and KEGG pathways in ASD and normal cells with reference gene sets c2.cp.kegg.v7.1.symbols.gmt and c5.go.bp.v7.2.symbols.gmt from MSigDB v7.1.^28^ The enrichment scores of each GO_BP term and KEGG pathway were calculated to obtain a score matrix, followed by differential analysis using the R package limma (version: 3.44.3). Results with *p*‐values <0.05 and |logFC| >0.263 were considered statistically significant.

### 2.9 Protein‐protein interaction (PPI) network and modules

The DEGs identified for each cell type were uploaded to the STRING database^29^ to investigate their interactions, using the following parameter: Homo sapiens and highest confidence (PPI score = 0.9). The PPI network was then visualized using Cytoscape^30^ (version 3.4.0). The CytoNCA plugin^31^ (Version 2.1.6) was used to analyse the degree of centrality for nodes in the PPI network without weighting. The MCODE plugin^32^ in the Metascape software was used to screen the key modules of the PPI networks using default parameters (Degree Cut‐off = 2, Node Score Cut‐off = 0.2, K‐core = 2, Max. Depth = 100). The modules with scores >5 were identified as key modules. ClusterProfiler^33^ (version:3.8.1) was used to investigate the KEGG pathways identified for the genes in key modules. A Benjamini and Hochberg adjusted *p*‐value <0.05 was used to identify significantly enriched pathways.

### 2.10 Immunohistochemical staining

Tissue sections were deparaffinized, rehydrated, and treated with citrate buffer (pH 6.0) to retrieve antigens. Then, 3% H_2_O_2_ was added to sections for 20 min to block endogenous peroxidase activity, and 3% bovine serum albumin was added to block nonspecific binding sites. The sections were incubated with primary antibody anti‐FABP4 antibody (Proteintech) and anti‐DCN antibody (Proteintech) at 4°C overnight, then incubated with secondary antibody at room temperature for 50 min. Diaminobenzidine was added as a chromogen and the sections were incubated for 2 h at room temperature. Sections were then counterstained with haematoxylin, rinsed, and air‐dried. Finally, the sections were sealed with neutral resin and examined under a fluorescent microscope (XSP‐C204; CIC). Three random fields were photographed under ×400 magnification and analysed using Image‐Pro Plus 6.0.

### 2.11 Cell culture and treatment

H9C2 cell lines were purchased from ATCC and cultured at 37°C with 5% CO_2_. The cells were seeded at a uniform density (10,000/cm^2^) and grown to 80% confluence in DMEM containing 10% foetal bovine serum and antibiotics. The medium was then replaced by DMEM without foetal bovine serum supplemented with ANP (MCE) for 12 h at different concentrations.

### 2.12 Annexin V‐FITC assay

Apoptotic capability was studied by staining the treated H9C2 cells with Annexin V‐FITC and Propidium Iodide (PI). The cells were collected after centrifugation, washed with phosphate‐buffered saline, and resuspended in Annexin buffer. The cells were then centrifuged at 700*g* for 5 min, the supernatant was discarded, and the pellet was resuspended in 1× binding buffer. Three‐hundred microliters of the sample solution was transferred to a 5 ml culture tube and incubated with 5 μl FITC‐conjugated annexin V (Meilune) and 5 μl PI (Meilune) for 15 min at room temperature in the dark. Two‐hundred microliters of 1× binding buffer was added to each sample tube, and the samples were analysed using a BD FACSVERSE flow cytometer with in‐built BD FACSuiteTM Software.

### 2.13 Small interfering RNA (siRNA) transfection

siRNAs were transfected into vascular smooth muscle cells (VSMCs) using Lipofectamine^®^ RNAiMAX (Invitrogen) according to the manufacturer's protocol. The siRNA for hsa_circ_0000280 was designed according to sequences of the junction point. All siRNAs were developed and synthesized by Shanghai GenePharma Co., Ltd, and their sequences are shown in Table S1.

### 2.14 RNA isolation and quantitative PCR (qPCR)

Total RNA from the tissue specimens and cells was isolated using TRIzol reagent (Life Technologies). To measure the levels of circRNA and mRNA, cDNAs were prepared using the Primescript RT Master Mix (Takara) and quantitative PCRs were carried out using TB Green Premix EX Taq (Takara). circRNA and mRNA expression were normalized to β‐actin levels using the 2^−ΔΔCT^ method. The primer sequences are shown in Table S2. The average cycle threshold for genes was calculated from a minimum of three separate measurements.

### 2.15 Statistical analysis

GraphPad PRISM 5 (Graphpad Software) was used for statistical analyses. ASD and normal samples were compared using the unpaired *t* test. *p*‐values <0.05 were considered statistically significant.

## 3 RESULTS

### 3.1 Identification of different cell types

**FIGURE 1:**
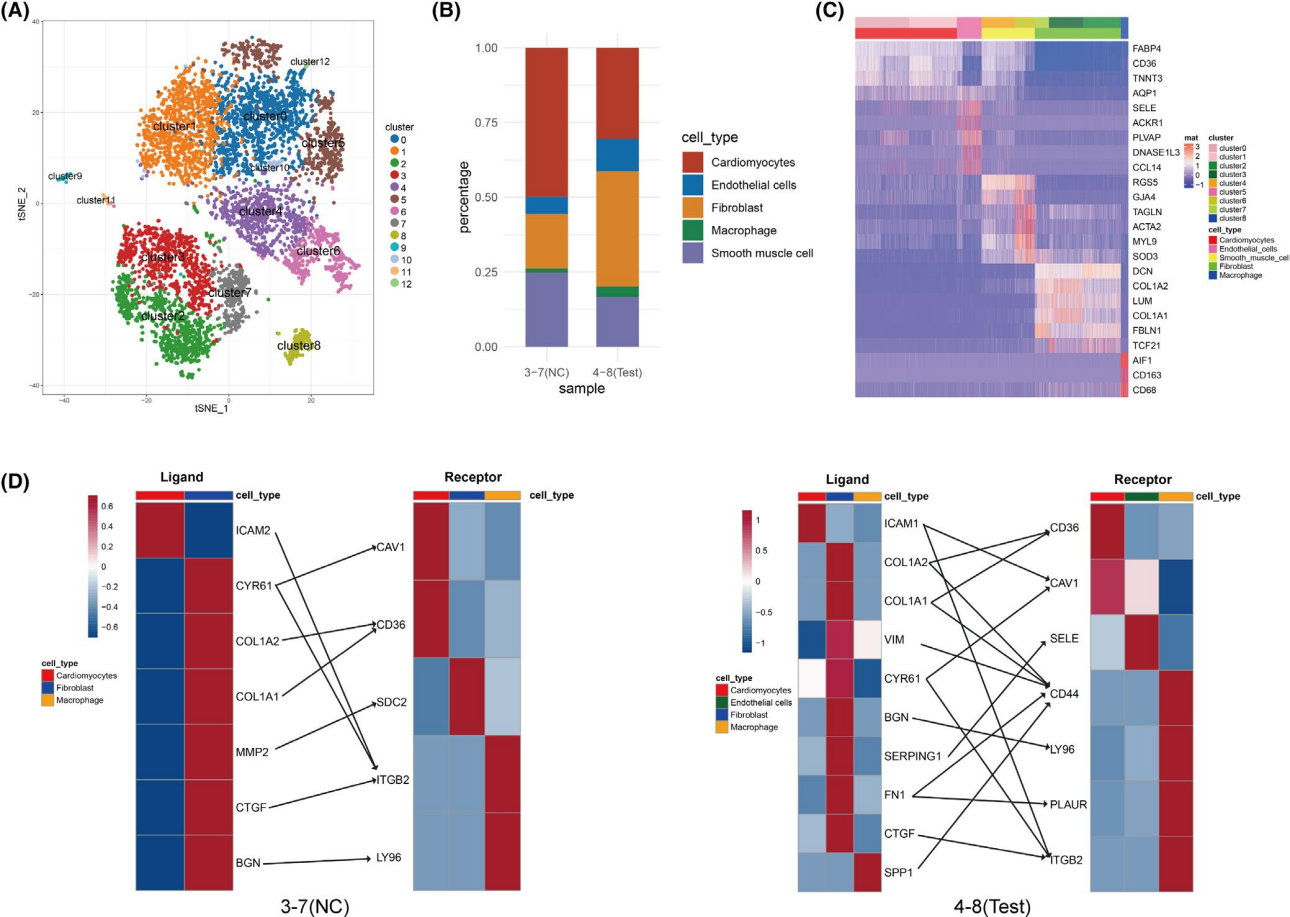
Cell clustering and cellular crosstalk analysis. (A) tSNE two‐dimensional distribution cluster of cells. Different colours represent different cell clusters. (B) Histogram showing the cell proportions in each cell type in ASD and normal samples. (C) Heatmap of marker genes across nine major cells clusters (Clusters 0–8). (D) Heatmap showing the potential ligand‐receptor pair expression (connected by straight lines) according to cell type in normal samples and ASD samples. 3–7 (NC), normal group; 4–8 (Test), ASD group

An expression matrix of 18,411 genes in 6487 cells was identified and used in this analysis. Unsupervised clustering showed that the 6,487 cells were clustered into 13 cell clusters (Figure 1A). After filtering the cell clusters with cell proportions less than 1%, there were nine cell clusters (clusters 0–8) retained and used in the following analysis (Table 1). As seen in Figure 1B, the cell proportions in each cell type were different in ASD and normal samples. The proportion of cardiomyocytes and smooth muscle cells were lower in ASD samples compared with normal samples, while ASD samples showed higher proportions of endothelial cells and fibroblast than the normal samples. In addition, the proportion of macrophages had also increased in ASD samples.

**TABLE 1:** Statistics of cells number and corresponding proportion

	number			Per cent (%)		
	3–7 (NC)	4–8 (Test)	Total	3–7 (NC)	4–8 (Test)	Total
cluster0	590	681	1271	25.911	15.8	19.296
cluster1	527	598	1125	23.144	13.875	17.079
cluster2	244	648	892	10.716	15.035	13.542
cluster3	114	687	801	5.007	15.94	12.16
cluster4	335	445	780	14.712	10.325	11.842
cluster5	127	453	580	5.578	10.51	8.805
cluster6	220	253	473	9.662	5.87	7.181
cluster7	51	281	332	2.24	6.52	5.04
cluster8	32	145	177	1.405	3.364	2.687
cluster9	9	43	52	0.395	0.998	0.789
cluster10	14	36	50	0.615	0.835	0.759
cluster11	8	24	32	0.351	0.557	0.486
cluster12	6	16	22	0.264	0.371	0.334

The expression of 24 marker genes is shown in Figure 1C. Cells in Clusters 0 and 1 showed significantly higher expression of FABP4, CD36, TNNT3 and AQP1, which are markers of cardiomyocytes. Therefore, Clusters 0 and 1 were considered cardiomyocyte clusters. Markers of endothelial cells, including SELE, ACKR1, PLVAP, DNASE1L3 and CCL14, were highly expressed in Cluster 5. Hence, Cluster 5 was defined as an endothelial cell cluster. Clusters 4 and 6 were considered smooth muscle cell clusters due to the high expression of markers RGS5, GJA4, TAGLN, ACTA2, MYL9 and SOD3. Markers of fibroblasts, including DCN, COL1A2, LUM, COL1A1, FBLN1 and TCF21, were highly expressed in Clusters 2, 3 and 7. Hence, Clusters 2, 3 and 7 were defined as fibroblast clusters. Cluster 8 was considered a macrophage cluster due to the high expression of markers AIF1, CD163 and CD68. Finally, five different cell types, cardiomyocytes, endothelial cells, smooth muscle cells, fibroblasts and macrophages were identified. The cardiomyocytes, endothelial cells, smooth muscle cells and fibroblasts contained different cell clusters, suggesting that these four cell types might contain different subpopulations of cells.

To strengthen our results, we also downloaded the public bulk data of GSE132176, GSE23959 and GSE35776 from GEO and extracted the samples we needed. Among them, samples of children with ASD were selected from GSE132176; Samples of neonates with right and left ventricles were selected from GSE23959 as normal controls. Control samples also were established in the right ventricle of infants extracted from GSE35776. Totally, 10 ASD cases and 18 controls were obtained. Based on markers we used for cell‐type identification, the ssGSEA was applied to explore the different infiltration degrees of cardiomyocytes and fibroblasts using the R package ‘GSVA’. As shown in Figure S1, it can be seen that in the ASD group, the infiltration level of cardiomyocytes has a significant decrease. In contrast, the infiltration level of fibroblasts has an obvious increase, which is basically consistent with our conclusion.

### 3.2 Ligand‐receptor interactions in cell crosstalk

Ligand‐receptor interactions among the cell types were investigated using iTALK. Most ligand‐receptor interactions were identified among cardiomyocytes, fibroblast and macrophage, suggesting that there was more cell crosstalk among these three cell types, especially more cell crosstalk between fibroblast and macrophage. From normal samples, 8 ligand‐receptor pairs were identified, including one growth factor ligand‐receptor interaction (fibroblast CTGF and macrophage ITGB2) and 7 other ligand‐receptor interactions. From ASD samples, 15 ligand‐receptor pairs were identified, including one growth factor ligand‐receptor interaction (fibroblast CTGF and macrophage ITGB2) and 14 other ligand‐receptor interactions (Figure 1D). Notably, growth factor ligand‐receptor interaction (fibroblast CTGF and macrophage ITGB2) was shared in normal and ASD samples. Interactions between fibroblast COL1A2/COL1A2‐cardiomyocyte CD36 were also common in both normal and SAD samples. Compared with normal samples, there was more cell crosstalk among cells in ASD samples. Most ligand‐receptor interactions were specific in normal samples (such as cardiomyocyte ICAM2‐macrophage ITGB2) and ASD samples (such as fibroblast BGN‐macrophage LY96).

We also analysed with a typical method CellPhoneDB. Ligand‐receptor interactions were identified in five cell types. Among them, there was more cell crosstalk among cardiomyocytes, fibroblast cells and macrophages in ASD sample (Figure S2). To show specific communication between them, we screen out 14 significant ligand and receptor gene pairs, displayed in a bubble graph (Figure S2). It was revealed that SPP1‐CD44 pairs were significantly activated in ASD samples, especially in macrophages. This point was consistent with the result in iTALK analysis. The bubble diagram proved that these ligands and receptors play essential roles in the crosstalk between cardiomyocytes, fibroblasts and macrophages.

### 3.3 Differences between ASD and normal samples for different cell types

**FIGURE 2:**
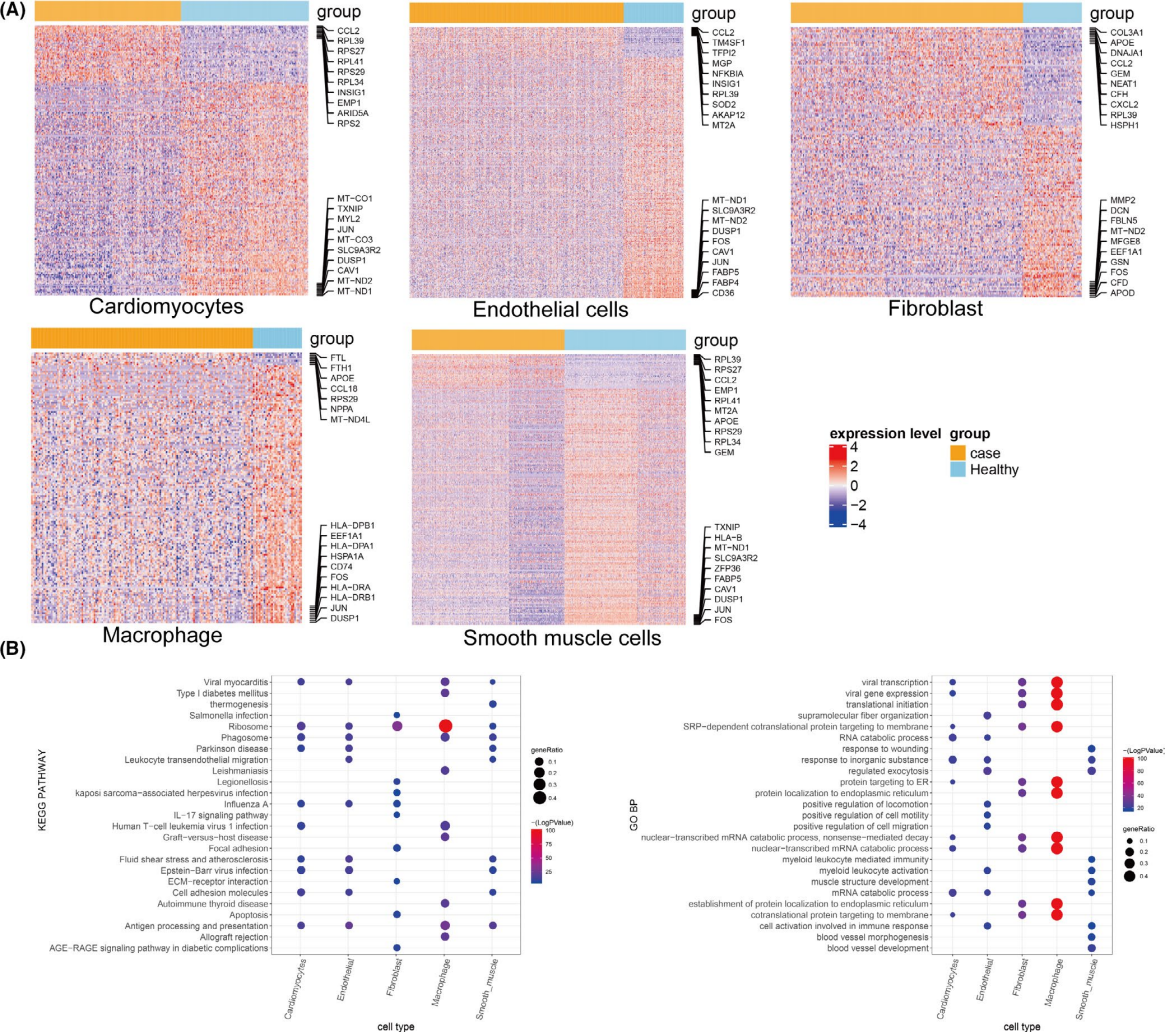
Heatmaps and functional enrichment analysis for DEGs. (A) Heatmaps showing DEGs in cardiomyocytes, endothelial cells, fibroblasts, macrophages and smooth muscle cell clusters between ASD and normal samples. The top 10 upregulated and downregulated genes are listed on right side. (B) Bubble diagram showing the top 10 GO_BP terms and KEGG pathways for the DEGs in the five cell types between ASD and normal samples. Vertical axes show enriched terms, and horizontal axes represent the genes in different cell clusters. Larger node size represents the larger ratio of enriched genes/total genes

We firstly examined the DEGs between ASD and normal samples in cardiomyocytes, endothelial cells, smooth muscle cells, fibroblasts and macrophages (Figure 2A and Figure S3). Endothelial cells showed most DEGs between ASD and normal samples. For all cell types, the majority of the DEGs were downregulated in ASD samples (Table 2).

**TABLE 2:** Statistics of DEGs between ASD and normal samples for different cell types

Cell type	Up	Down	Total
Cardiomyocytes	42	157	199
Endothelial cells	35	275	310
Smooth muscle cell	32	218	250
Fibroblast	57	100	157
Macrophage	7	141	148

For cardiomyocytes, there were 199 DEGs between ASD and normal samples, including 42 upregulated genes and 157 downregulated genes. These DEGs were mainly enriched for some RNA‐related biological processes, such as RNA catabolic process, nuclear‐transcribed mRNA catabolic process (nonsense‐mediated decay), and nuclear‐transcribed mRNA catabolic process, and KEGG pathways such as viral myocarditis, cell‐adhesion molecules and regulation of lipolysis in adipocytes (e.g., FABP4, NPPA and MGLL). There were similarities and differences in the functional enrichment results for DEGs of different cell types. The DEGs for fibroblast were primarily involved in biological processes, such as translational initiation and protein localization to the endoplasmic reticulum, and in KEGG pathways, such as focal adhesion, ECM‐receptor interaction and apoptosis. DEGs in macrophages were significantly enriched in antigen processing and presentation. The DEGs for smooth muscle cells were implicated in blood vessel morphogenesis and development (Figure 2B).

### 3.4 Gene set variation analysis

According to the methods described above, GSVA was performed to evaluate the differences in GO_BP terms and KEGG pathways between ASD and normal samples for each cell type. Six KEGG pathways showed significant differences between ASD and normal samples for cardiomyocytes. For example, ribosome was enriched in ASD, while antigen processing and presentation was significantly enriched in normal samples. A total of 8, 6, 0 and 9 KEGG pathways with significant differences were found for endothelial cells, smooth muscle cells, fibroblasts and macrophages, respectively (Figure S4).

**FIGURE 3:**
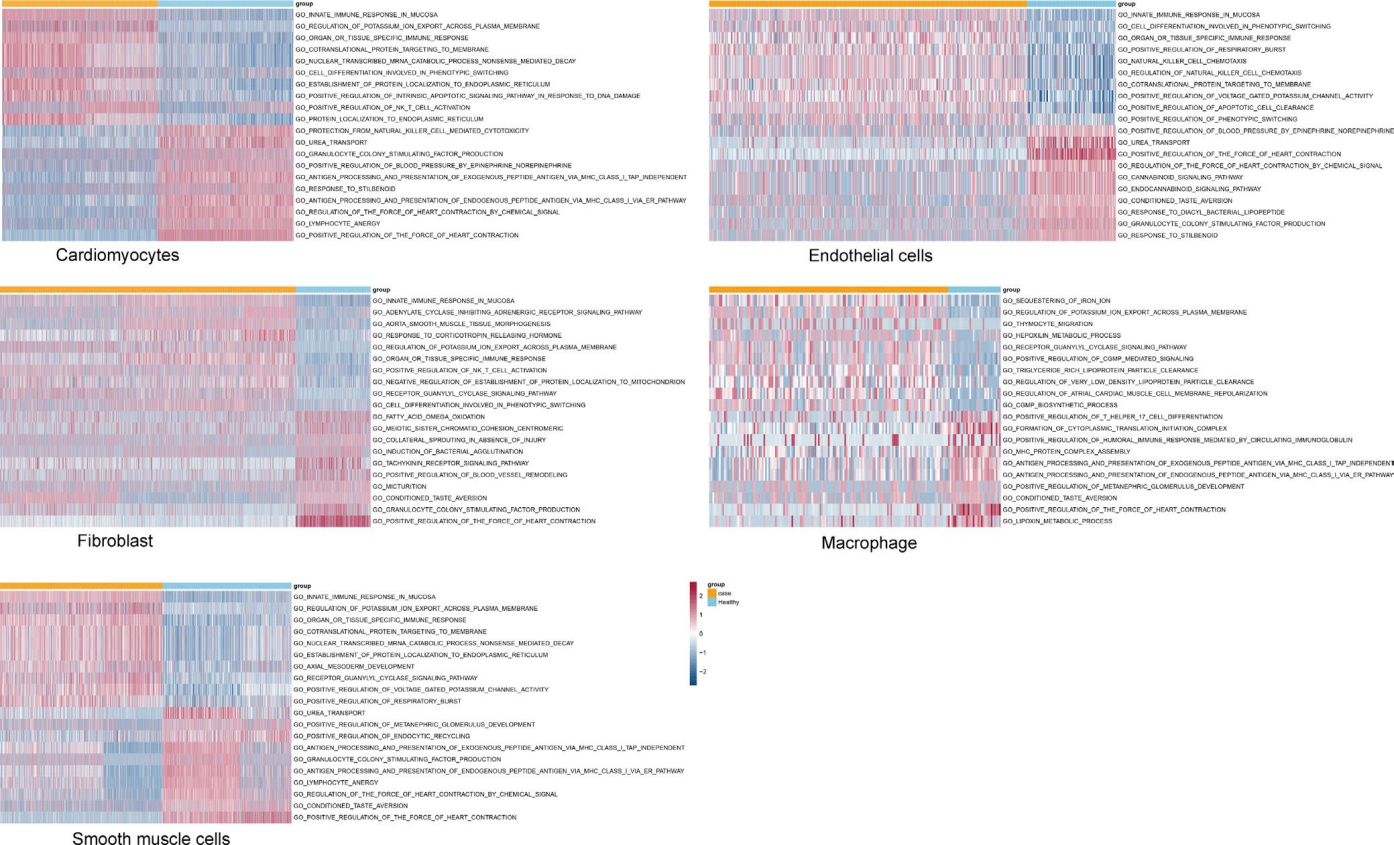
Differential biological processes in gene set variation analysis (GSVA). Heatmaps showing the differential biological processes in the GSVA analysis for the five cell types between ASD and normal samples

A total of 191 GO_BP terms showed significant differences between ASD and normal samples for cardiomyocytes. For example, innate immune response in mucosa and regulation of potassium ion export across the plasma membrane were significantly enriched in ASD samples, while positive regulation of the force of heart contraction was significantly enriched in normal samples. There were 242, 177, 75 and 132 GO_BP terms with significant differences for endothelial cells, smooth muscle cells, fibroblasts and macrophages, respectively. For example, aorta smooth muscle tissue morphogenesis was associated with ASD in fibroblasts. For endothelial cells, natural killer cell chemotaxis was significantly enriched in ASD samples (Figure 3).

### 3.5 PPI network and module analysis for each cell type

**FIGURE 4:**
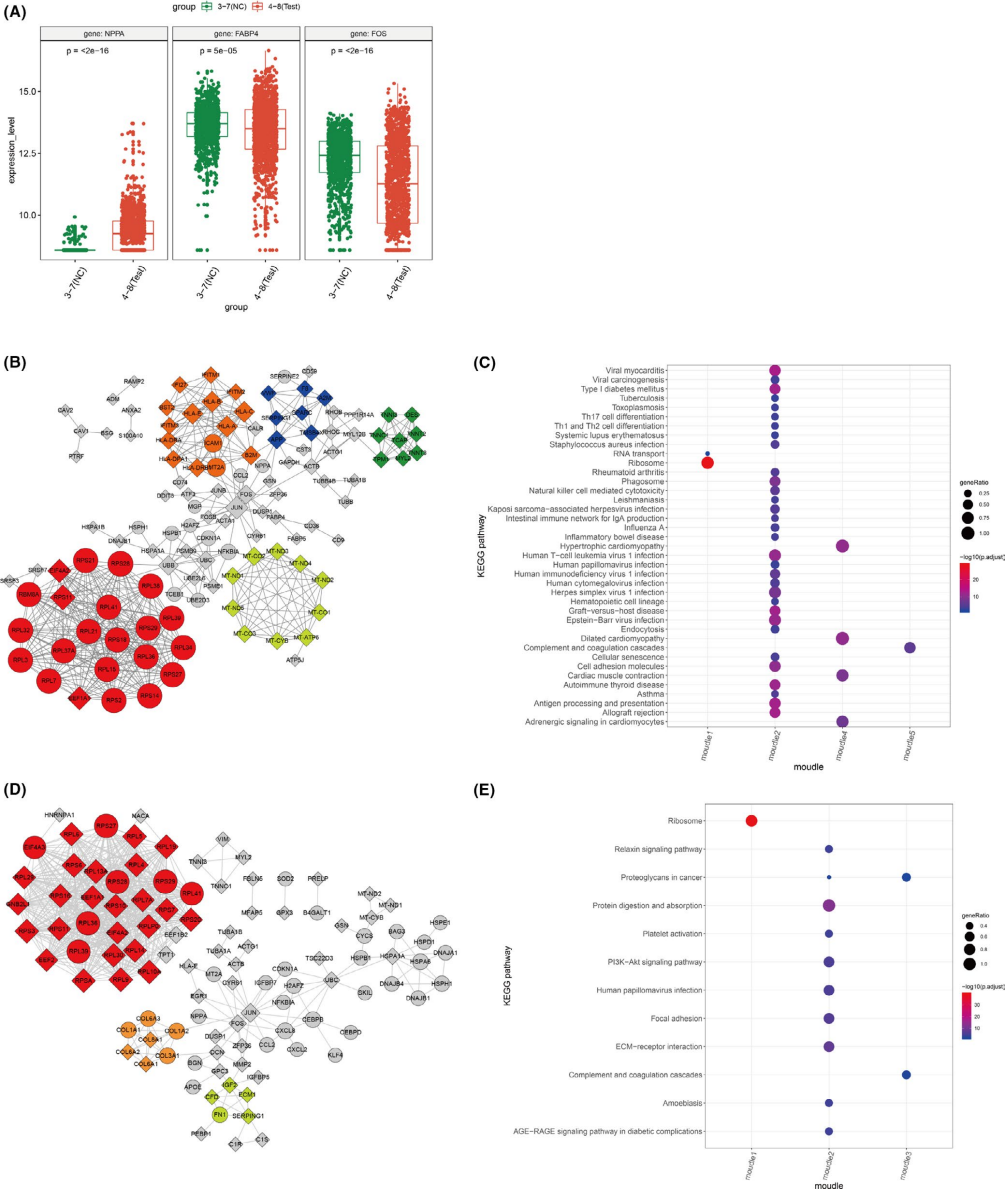
PPI network and module analysis for DEGs in cardiomyocytes and fibroblasts. (A) Boxplots showing the expression of FABP4, FOS and NPPA in cardiomyocytes between normal and ASD samples; (B and D) PPI network showing the interactions among DEGs in cardiomyocytes and fibroblasts. Circle nodes represent upregulated genes, and rhombus nodes represent downregulated genes. Nodes with the same colour represent the genes in one module (score >5). Red module, module 1; orange module, module 2; yellow module, module 3; green module, module 4; blue module, module 5. (C and E) Bubble diagrams showing the significantly enriched KEGG pathways for genes in each module

We also investigated the interactions among these DEGs (Table 3). For cardiomyocytes, the PPI network contained 121 nodes and 519 interactions. Among these interactions, cardiomyocyte marker gene FABP4 showed interaction with FOS (c‐fos), while FOS showed interaction with NPPA (ANP), which has been reported to play an important role in heart development.^34^, ^35^ Compared to normal samples, expression of FABP4 and FOS were decreased in ASD samples, while expression of NPPA was increased in ASD samples (*p* < 0.05) (Figure 4A). A total of five modules were identified from the PPI network (Figure 4B). Genes in different modules were enriched for different pathways. For example, genes in module 1 (red module) were implicated in the ribosome pathway, and genes in module 5 (blue module) were enriched for the complement and coagulation cascade pathways (Figure 4C).

**TABLE 3:** Statistics of nodes and interactions in PPI network for different cell types

Cell type	Node	Edge	Module(score ≥5)
Cardiomyocytes	121	519	5
Endothelial cells	190	919	8
Smooth muscle cell	149	552	6
Fibroblast	107	606	3
Macrophage	124	2659	3

For fibroblasts, the PPI network contained 107 nodes and 606 interactions. The fibroblast marker gene DCN showed interaction with MMP2, which has been reported to play an important role in the regulation of myocardial extracellular matrix homeostasis and cardiac remodelling.^36^, ^37^, ^38^ Three modules were identified from the PPI network (Figure 4D). Genes in different modules were enriched for different pathways. Similarly, genes in module 1 (red module) were implicated in the ribosome pathway, and genes in module 3 (yellow module) were enriched in the complement and coagulation cascade pathways. Genes in module 2 (orange module) were enriched for various pathways, such as protein digestion and absorption, and ECM‐receptor interaction (Figure 4E). In addition, there were more interactions among DEGs for macrophages, and the PPI network contained 124 nodes and 2,659 interactions. The PPI networks for macrophages, endothelial cells, and smooth muscle cells are shown in Figure S5. Similarly, DEGs in different modules were significantly enriched for different pathways (Figure S5).

### 3.6 Cell pseudotime trajectory analysis

**FIGURE 5:**
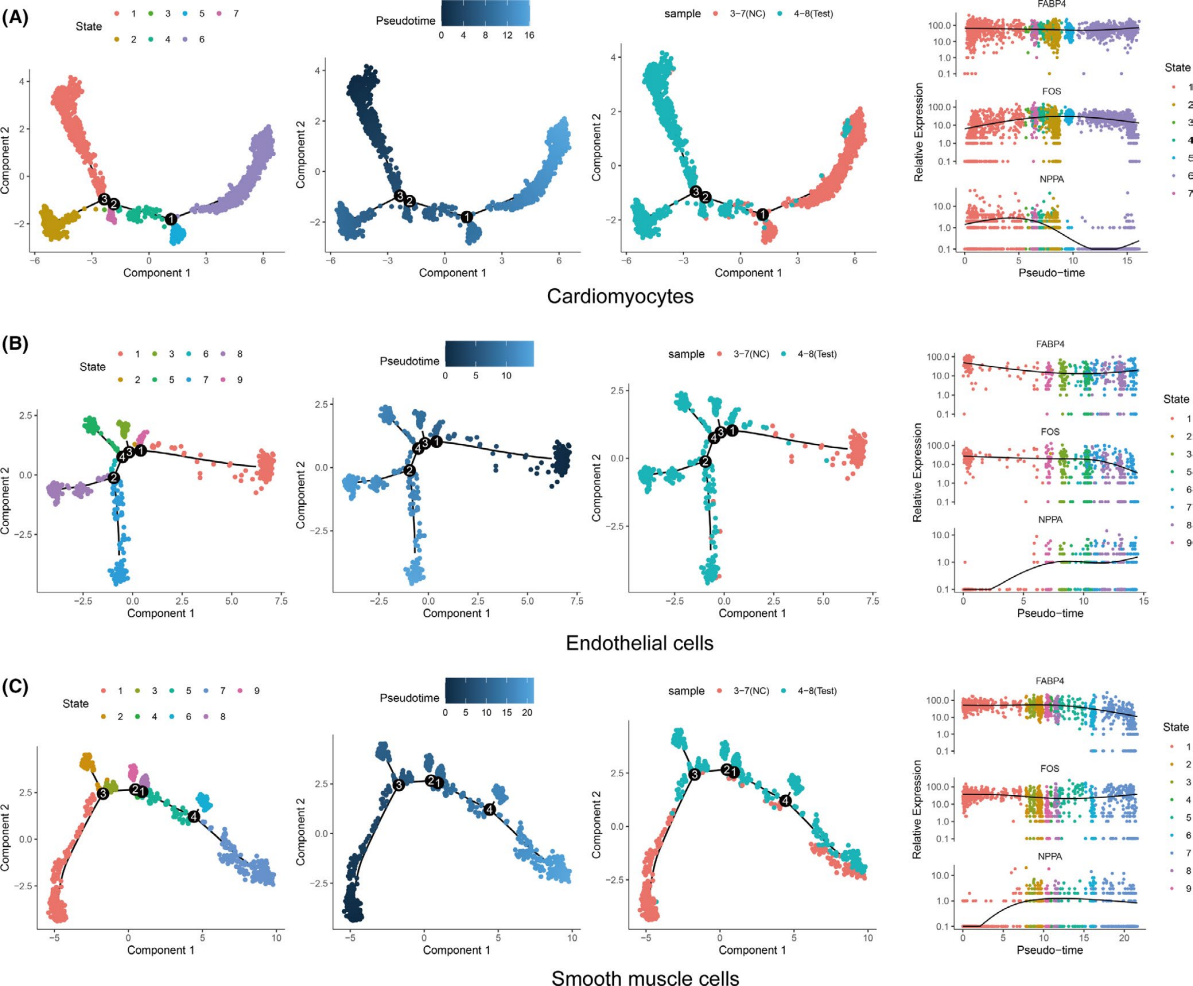
Pseudotime analysis of gene expression in ASD and normal. (A) Cardiomyocytes trajectories by state, pseudotime and groups; expression changes of FABP4, FOS and NPPA along the pseudotime (right side). (B) trajectories of endothelial cells by state, pseudotime and groups; expression changes of FABP4, FOS and NPPA along the pseudotime (right side); (C) trajectories of smooth muscle cell by state, pseudotime and groups; expression changes of FABP4, FOS and NPPA along the pseudotime (right side)

We also performed cell trajectory analysis using Monocle to order individual cells in pseudotime for cardiomyocytes, endothelial cells and smooth muscle cells, respectively. As shown in Figure 5, cells from normal tissues and ASD tissues were distributed in different trajectory states for all three cell types, suggesting there were significant difference between normal and ASD samples. Cardiomyocytes transition from normal to disease tended to decrease by pseudotime, while colorectal cells and Smooth muscle cells transition from normal to disease tended to increase by pseudotime. Figure 5A showed the pattern of pseudotime trajectory for cardiomyocytes, cell from normal tissue and ASD tissues mainly distributed in both ends of the pseudotime branch. Expression pattern FABP4 were relatively stable along the pseudotime. Expression of FOS was increased along the pseudotime, with an increase at state 5 and 6 (cells from normal tissues), while expression of NPPA was decreased along the pseudotime, especially at state 5 and 6 (cells from normal tissues). Figure 5B showed the pattern of pseudotime trajectory for endothelial cells. Cells from normal tissue are mainly distributed at the initiation of pseudotime branching, and cells from ASD tissue are distributed at different pseudotime branching. Expression of FABP4 and FOS showed a decrease along the pseudotime, suggesting a decreased level in ASD tissue, while NPPA showed an increase expression in ASD tissue. Figure 5C showed the pattern of pseudotime trajectory for smooth muscle cells. Cells from normal tissue distributed along the pseudotime, mainly at both the initiation and end of pseudotime branching. At the end of pseudotime branching (state 7), normal samples and ASD samples showed equal proportions. FABP4 and FOS showed relatively stable expression, while NPPA showed an increase expression along the pseudotime. In general, through pseudotime trajectory analysis, we successfully constructed the cell trajectory in ASD and identified the trajectory change process of key genes in different states. These findings provide a basis for us to thoroughly understand the important regulatory role of different genes in the cellular changes in ASD.

### 3.7 Immunohistochemical staining

**FIGURE 6:**
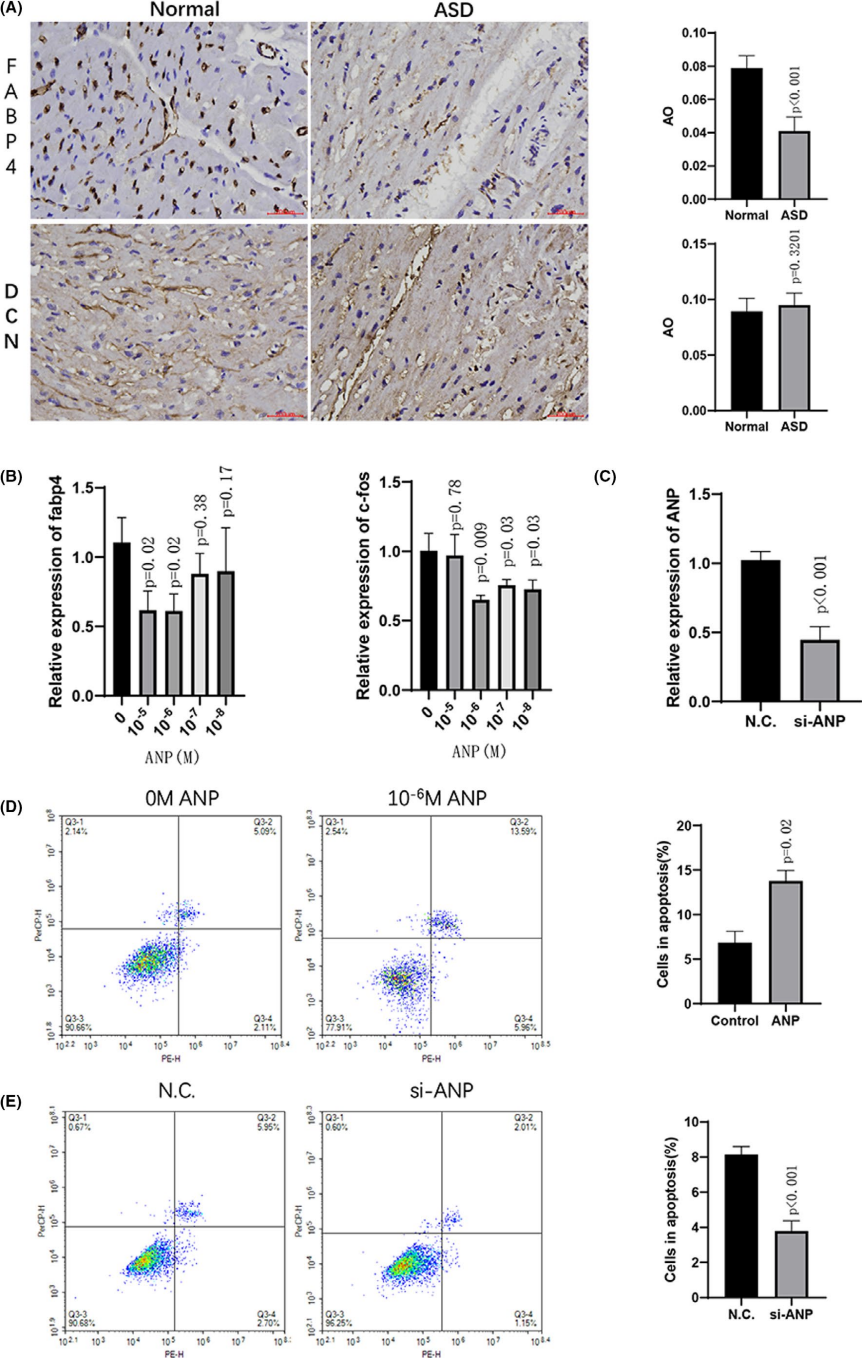
Immunohistochemical staining and in vitro assay. (A) Representative images (400×) and quantitative statistics of protein expression of DCN (fibroblast marker) and FABP4 (cardiomyocyte marker) determined by immunohistochemical staining. Scale bar, 200 µm; *n* = 9, **p < 0.01, compared with the normal group. (B) Expression of FABP4 and FOS (c‐fos) was measured in ANP‐induced cells at different concentrations for 12 h. *n* = 3, **p < 0.01, *p < 0.05, compared with 0 M ANP. (C) The expression of Nppa (ANP) was measured after the transfection of si‐ANP; *n* = 3, **p < 0.01, compared with the negative control (N. C.). (D) representative images and quantitative statistics of 10^−6^ M ANP‐induced apoptosis by annexin V‐FITC assay; *n* = 3, **p < 0.01, compared with 0 M ANP. (E) representative images and quantitative statistics of annexin V‐FITC assay in ANP knockdown study; *n* = 3, **p < 0.01, compared with the negative control (N. C.)

A proportion of cardiomyocytes were decreased in ASD samples compared with normal samples, while ASD samples showed a higher proportion of fibroblasts than normal samples. Therefore, cardiomyocytes and fibroblasts were considered important cell types. We determined marker gene expression by immunohistochemical staining for these two cell types. As shown in Figure 6A, the expression of DCN (fibroblast marker) showed a trend towards an increase without statistical significance in the ASD group compared with the normal group. The expression level of FABP4 (cardiomyocyte marker) was significantly decreased in the ASD group compared to the normal group, which indicates a relatively smaller number of cardiomyocytes in the ASD group compared to the normal group. We then performed ANP dose‐dependent stimulation from 10^−8^ M to 10^−5^ M in H9C2 cells. ANP‐induced stimulation decreased the expression of FABP4 at the concentration of 10^−5^ M and 10^−6^ M, while the c‐Fos level was reduced at concentrations from 10^−8^ M to 10^−6^ M (Figure 6B). Therefore, the stimulation of 10^−6^ M ANP induced the reduction of both FABP4 and c‐Fos, which was consistent with the result from single‐cell sequencing analysis. The annexin V‐FITC assay showed that ANP stimulation also aggravated the apoptosis of cardiomyocytes (Figure 6D). Further, we designed siRNA for ANP and remarkably reduced the expression of ANP in H9C2 cells (Figure 6C). The knockdown of ANP decreased the percentage of cells in apoptosis (Figure 6E).

## 4 DISCUSSION

The heart is the central organ of the circulatory system, and its normal development plays an important role in sustaining life. ASD is a serious cardiac problem but understanding of the pathogenesis and pathways of this disease is still limited. In this study, we performed a comprehensive study of single‐cell RNA sequencing data from cells derived from ASD and normal control tissues. A total of 18,411 genes in 6587 cells were investigated, and five major cell types were identified, including cardiomyocytes, endothelial cells, fibroblasts, macrophages and smooth muscle cells.

Compared to normal tissue, the proportion of cardiomyocytes significantly decreased, while the proportion of fibroblasts significantly increased in ASD tissue. Immunohistochemical staining of fibroblast markers and cardiomyocyte markers also confirmed this finding. Cui et al. tracked the development of the human heart by single‐cell transcriptome analysis and suggested that cardiomyocytes make up the largest population in the human foetal heart. Their second‐level clustering results revealed that cardiomyocytes are mainly distributed in the ventricles and atria.^22^ In addition, Jie et al. observed cardiomyocyte apoptosis in all recruited ASD patients, but not in controls.^39^ In addition, ASD leads to chronic atrial stretching, and this mechanical stretch causes elevated expression of AngII and TGF‐beta 1, as well as collagen synthesis in cardiac fibroblasts. It also stimulates cardiomyocyte signalling by activating angiotensin II type 1 (AT1) receptors and mitogen‐activated protein kinase, with direct fibroblast‐activating effects,^40^, ^41^, ^42^ which leads to a lower percentage of atrial cardiomyocytes, and a larger concentration of fibroblasts.^43^ In addition, ligand‐receptor interaction analysis showed that there was more cell crosstalk among cardiomyocytes and fibroblasts, including cardiomyocyte HSPG2‐fibroblast PTPRS, cardiomyocyte A2 M‐fibroblast LRP1 and fibroblast COL1A2‐cardiomyocyte CD36. This also explained the importance of cardiomyocytes and fibroblasts in ASD. Therefore, we speculated that apoptosis of cardiomyocytes and an increase in fibroblasts might play an important role in the pathogenesis of ASD.

We further investigated the differences in molecular expression between ASD and normal samples for the five cell types. Endothelial cells showed most DEGs, while macrophages showed less DEGs between ASD and normal samples. There were similarities and differences in the functional enrichment results of DEGs for these five cell types. The DEGs for cardiomyocytes were primarily involved in viral myocarditis, cell adhesion molecules, and regulation of lipolysis in adipocytes (e.g., FABP4, NPPA, etc.). The DEGs for fibroblasts were primarily involved in focal adhesion, ECM‐receptor interaction and apoptosis. The DEGs for smooth muscle cells were implicated in blood vessel morphogenesis and development.

For cluster 9–12 with cell proportions less than 1%, we also use the R package ‘Seurat’ to analyse marker genes. Then, Gene Ontology (GO_BP) terms and KEGG pathways were analysed for DEGs identified in each cell type using the online Metascape tool. After the term that meets the above parameters is obtained, further clustering is carried out according to the genetic similarity (similarity of >0.3) in each term, and the most significant term (*p*‐value) in the cluster is selected to represent the cluster. The top 20 terms (ranked by *p*‐value) were displayed in bar diagram (Figure S6). Although it's hard to identify the definite information about the type of cells. According to GO and KEGG analysis, we inferred that Cluster 9 may participate in immune‐related response, and Cluster 11 functions as nervous system regulation. Cluster 10 located in the crowd of cardiomyocytes, endothelial cells and smooth muscle cells, with the function of angiogenesis and cell‐substrate adhesion. Cluster 12 was nearby Cluster 0 and 1 (cardiomyocytes), indicating that they may have certain similarities. GO analysis enhanced the inference that Cluster 12 was related to muscle system regulation.

Cardiomyocyte marker gene FABP4 showed interaction with FOS, while FOS showed an interaction with NPPA, which has been reported to play an important role in heart development.^34^, ^35^ NPPA (Natriuretic Peptide A) is an important gene in heart development and encodes proteins that belong to the natriuretic peptide family. Proteins of this family play an important role in the mediation of cardio‐renal homeostasis and are implicated in vascular remodelling and the regulation of energy metabolism. In addition, studies have also demonstrated that NPPA is implicated in the inhibition of cardiac remodelling and cardiac hypertrophy by inducing cardiomyocyte apoptosis and reducing the growth of cardiomyocytes and fibroblasts.^34^, ^44^ Cardiomyocytes express FABP4 (fatty acid‐binding protein 4), which is implicated in the regulation of heart function and directly contributes to cardiac metabolism and physiopathology.^45^ High expression of FABP4 promotes the development of cardiac hypertrophy by activating ERK signalling.^46^ In addition, FABP4 was found to be detrimental to cardiomyocyte survival. Sun et al. showed that inhibition of FABP4 could protect cardiomyocytes from apoptosis caused by hypoxia.^47^ These genes were all differentially expressed in ASD and normal samples in this study. Similarly, in‐vitro assays have shown that Nppa participates in the regulation of cardiomyocyte apoptosis, with the altered expression of FABP4 and FOS. Therefore, we conclude that these genes might be implicated in the physiopathology of ASD development.

There are some previously reported ASD related biomarkers, such as ACTC1, Alk3 and Whsc1. Alpha‐cardiac actin (ACTC1), which is essential for cardiac contraction, has been reported that reduced ACTC1 levels may lead to ASD.^48^, ^49^ We found that ACTC1 was downregulated in cardiomyocytes, endothelial cells, fibroblast cells and smooth muscle cells of ASD samples in our sequencing data (Figure S7). BMP receptor Alk3 plays an essential role in BMP signalling, which may contribute to human congenital heart diseases. In our results, Alk3 (BMPR1A) was downregulated in endothelial cells (Figure S7). It has been reported that conditional endothelial depletion of Alk3 severely impairs cushion morphogenesis during mammalian cardiogenesis.^50^ Alk3‐mediated BMP signalling is required for endocardial formation and survival of AV cushion mesenchymal cells.^51^ These pieces of evidence indicated that Alk3 induced endothelial dysfunction might be a key reason for ASD formation. Wolf–Hirschhorn Syndrome Candidate 1 (Whsc1) deletion of mice showed various atrial and ventricular septal defects.^52^ It is consistent with our result that Whsc1 was downregulated in cardiomyocytes, endothelial cells, and smooth muscle cells (Figure S7). Whsc1 can interact with Nkx2.5 to repress transcription of NKX2‐5 target genes such as Nppa. Nppa is aberrantly expressed in Whsc1 deleted hearts. Our results also confirmed the change of Nppa in ASD. Therefore, our study proved these reported conclusions and provided a new viewpoint of single cell.

Our study had some limitations. The major limitation is the small sample size, which could have led to large batch effects, and the reliability of the results may have been reduced to some extent. However, collecting human cardiac tissue poses great challenges in China, as infants’ and children's bodies are rarely donated for scientific research. The lack of human tissue that contains disease characteristics limits scRNA‐seq investigation of human disease, including ASD. This was not the only challenge during our study. In addition, studies of heart development using scRNA‐seq have mostly been based on animal models and less on human cardiac tissue. Furthermore, normal atrial septal tissue should ideally be used as a normal control. However, the patient had a serious atrial septal defect, and ventricular muscle tissue was collected as the normal control, considering that the lesion might affect atrial muscle. Only one marker gene for cardiomyocytes and fibroblasts was investigated by immunohistochemical staining. We preliminarily analysed several genes in cardiomyocytes. Further experiments should be carried out on different cell types.

In conclusion, we characterized cell subsets in ASD and normal samples, and five major cell types, including cardiomyocytes, endothelial cells, fibroblasts, macrophages and smooth muscle cells, were identified. ASD samples showed a decreased proportion of cardiomyocytes and an increased proportion of fibroblasts, and there was more cellular crosstalk between cardiomyocytes and fibroblasts. There were similarities and differences in DEGs and their functions between ASD and normal samples for these cell types. These findings increase the understanding of the underlying mechanisms of ASD at both the cellular and molecular level, and highlight potential targets for the treatment of ASD.

## CONFLICT OF INTEREST

None declared.

## AUTHOR CONTRIBUTIONS

**Zunzhe Wang:** Data curation (equal); Investigation (equal); Methodology (equal); Resources (equal); Software (equal); Supervision (equal); Writing‐original draft (equal); Writing‐review & editing (equal). **Huating Wang:** Supervision (equal); Validation (equal); Visualization (equal). **Ya Zhang:** Validation (equal); Visualization (equal). **Fangpu Yu:** Software (equal); Supervision (equal); Validation (equal). **Liwen Yu:** Investigation (equal); Methodology (equal). **Cheng Zhang:** Conceptualization (equal); Funding acquisition (equal).

## Supporting information

Fig S1

Fig S2

Fig S3

Fig S4

Fig S5

Fig S6

Fig S7

Table S1‐2

## Data Availability

The data that support the findings of this study are available from the corresponding author upon reasonable request.
