# Supplementary figures and images for: Single‐cell RNA sequencing analysis to characterize cells and gene expression landscapes in atrial septal defect
Source: J Cell Mol Med. 2021 Sep 12;25(20):9660–73. doi: 10.1111/jcmm.16914 (PMC8505850; doi:10.1111/jcmm.16914)

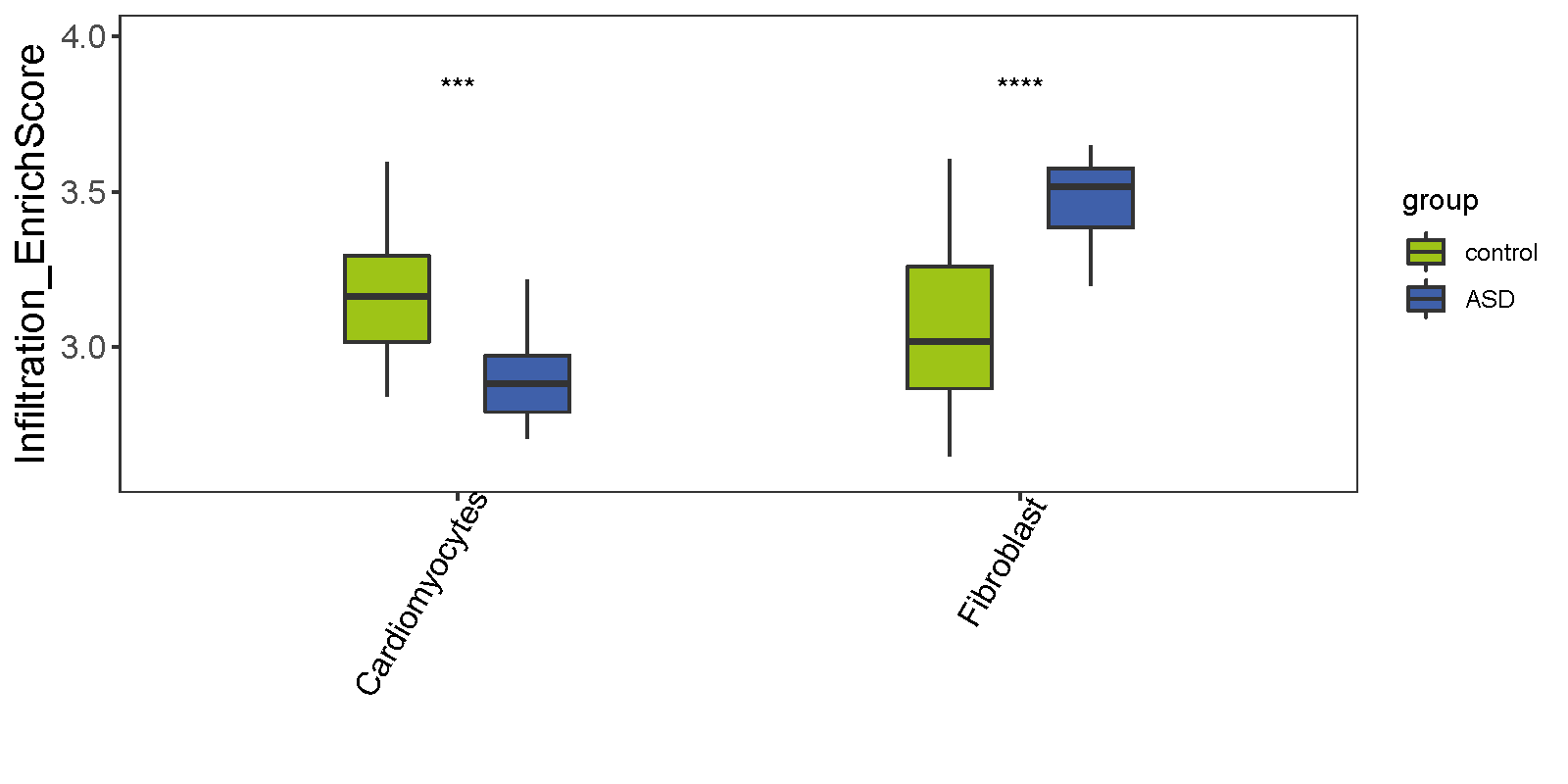

Supplement: Supplementary file 1 — Fig S1 [file JCMM-25-9660-s006.tif]

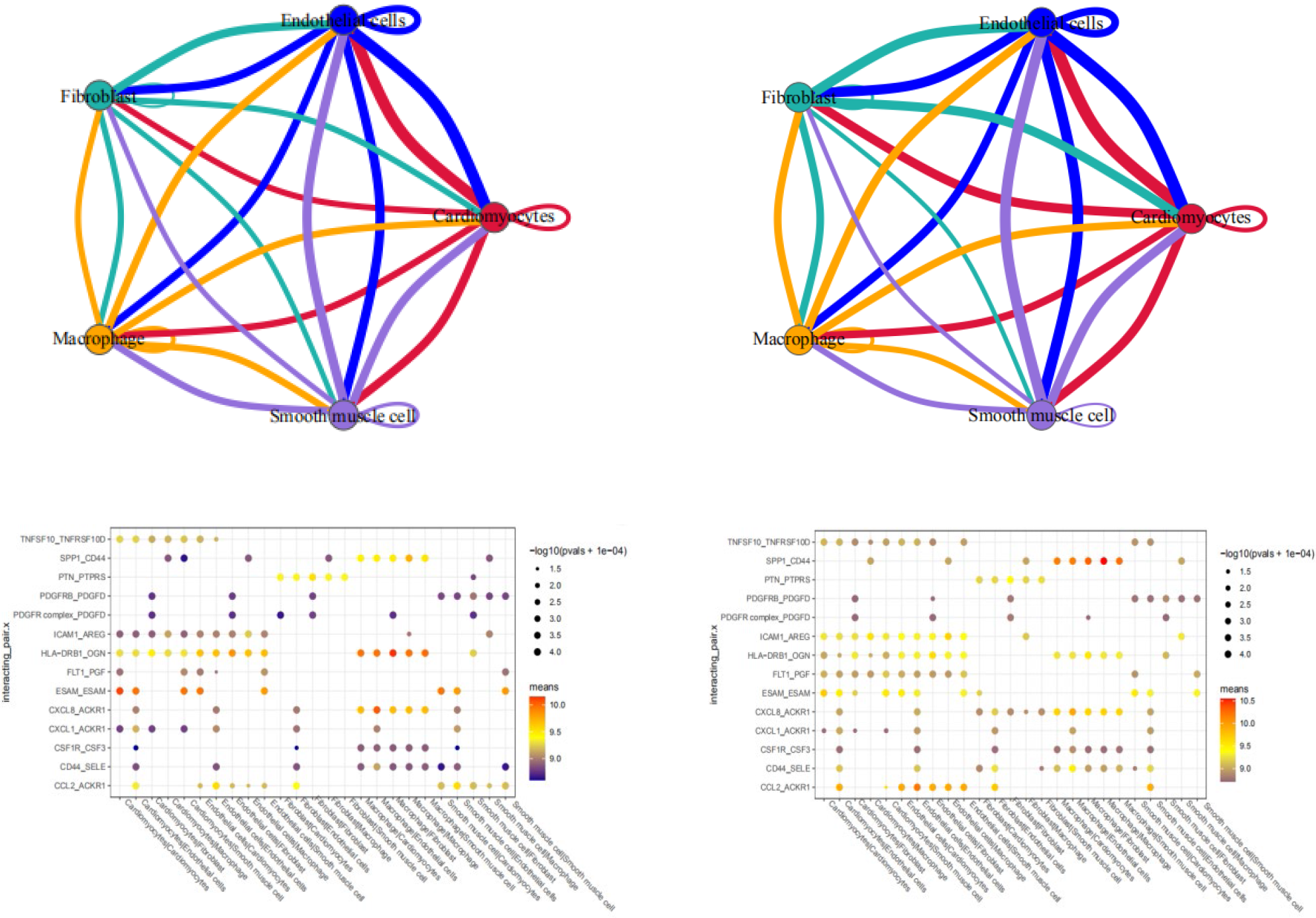

Supplement: Supplementary file 2 — Fig S2 [file JCMM-25-9660-s005.tif]

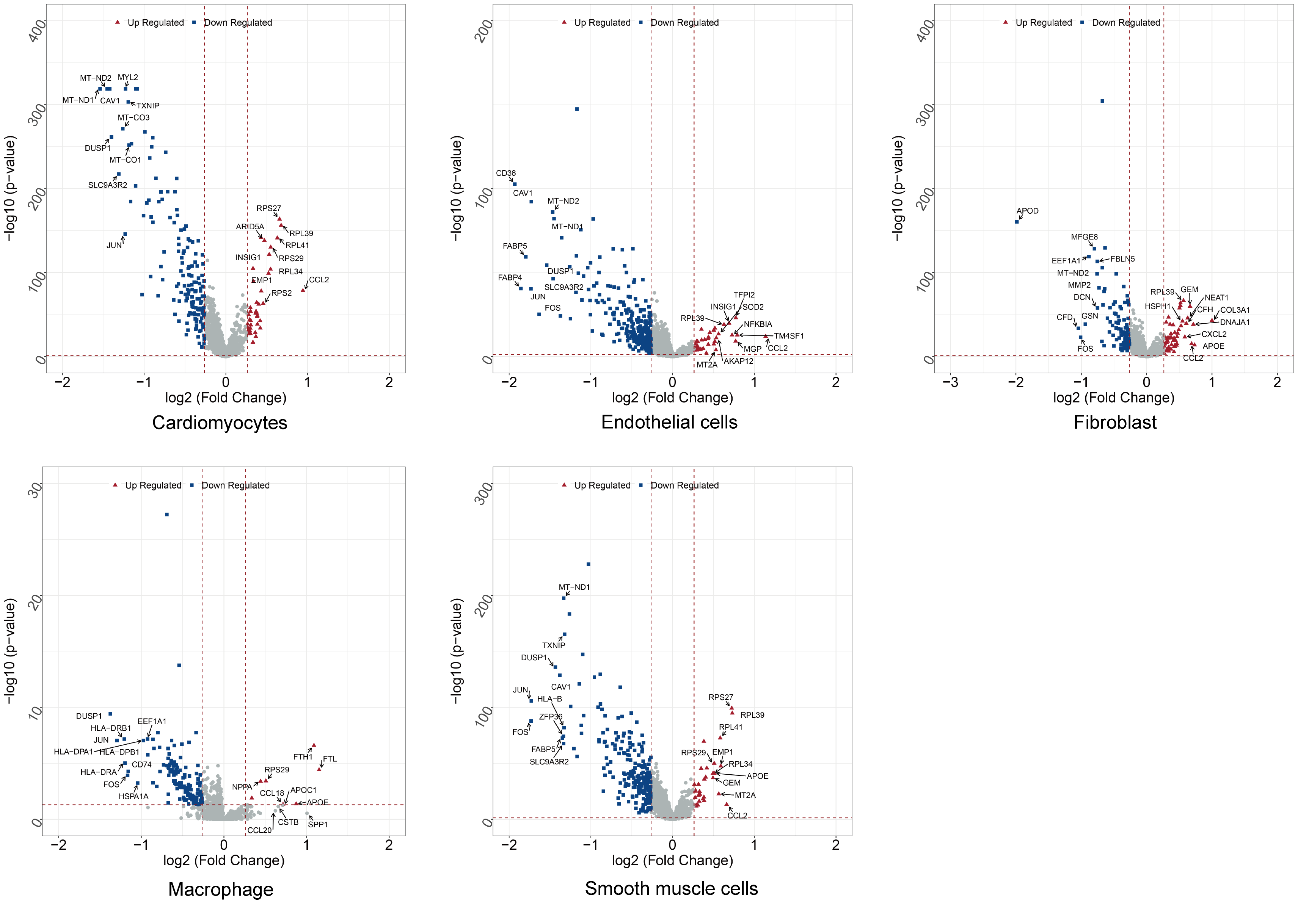

Supplement: Supplementary file 3 — Fig S3 [file JCMM-25-9660-s007.tif]

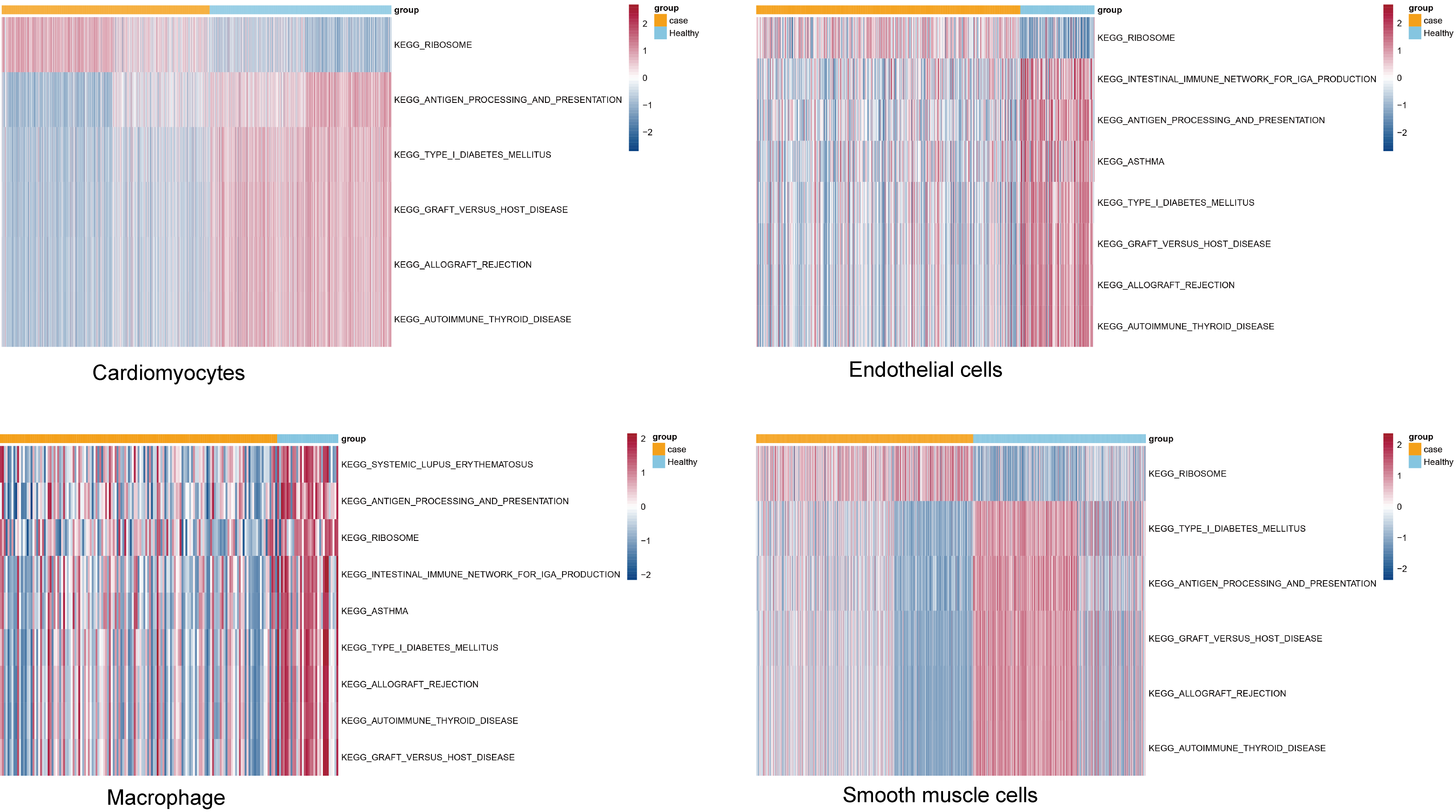

Supplement: Supplementary file 4 — Fig S4 [file JCMM-25-9660-s008.tif]

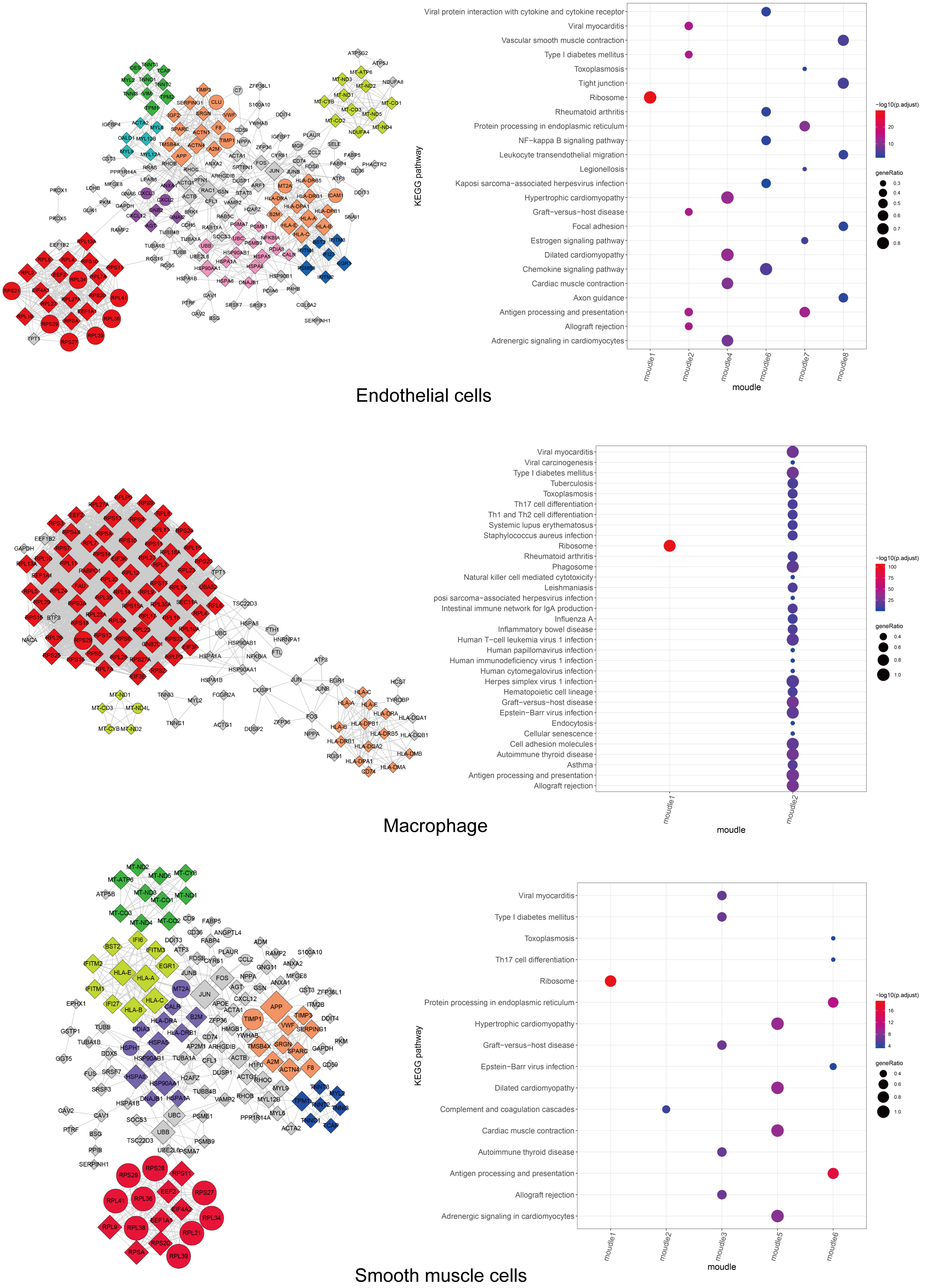

Supplement: Supplementary file 5 — Fig S5 [file JCMM-25-9660-s001.tif]

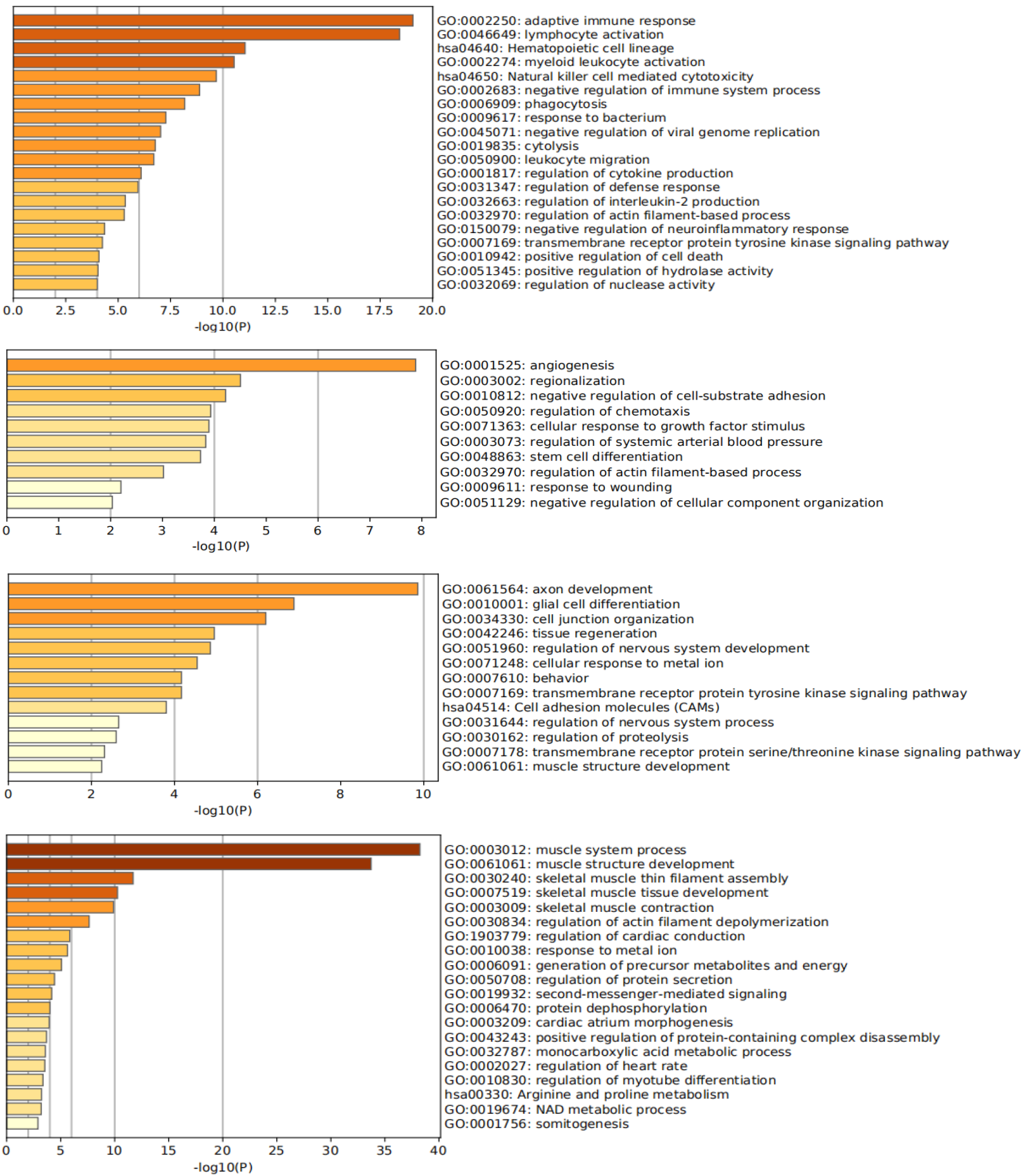

Supplement: Supplementary file 6 — Fig S6 [file JCMM-25-9660-s003.tif]

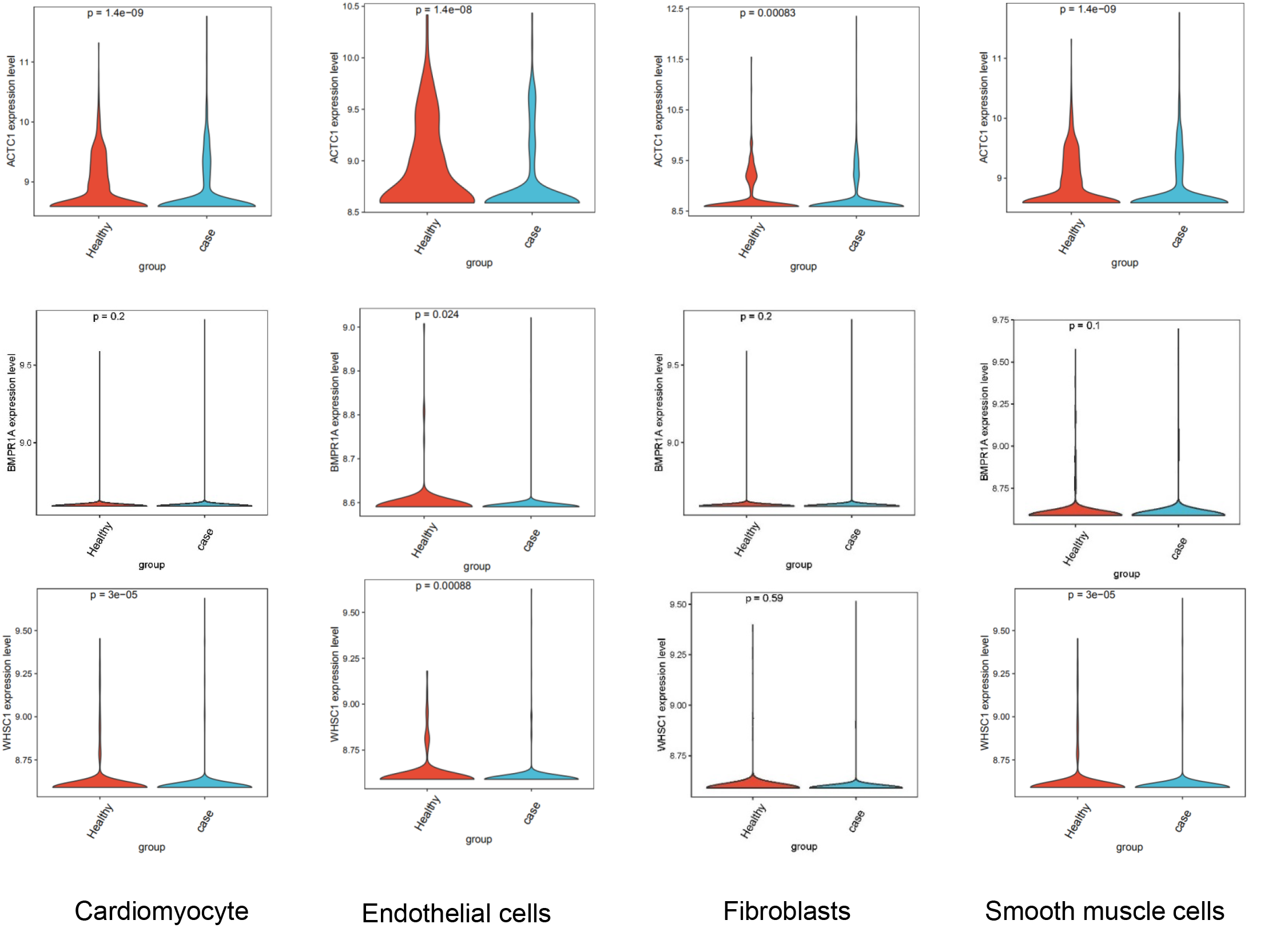

Supplement: Supplementary file 7 — Fig S7 [file JCMM-25-9660-s002.tif]
