# Supplementary material for: Single‐cell RNA sequencing analysis to characterize cells and gene expression landscapes in atrial septal defect
Source: J Cell Mol Med. 2021 Sep 12;25(20):9660–73. doi: 10.1111/jcmm.16914 (PMC8505850; doi:10.1111/jcmm.16914)
Supplement: Supplementary file 8 — Table S1‐2 [file JCMM-25-9660-s004.docx]

Table S1. The upstream and downstream of ANP siRNA

| Primer | | Sequence |
| --- | --- | --- |
| 1^st^ siRNA sense | 5’-CUAUCACGAUCGAUGUUAAUU-3’ | |
| 1^st^ siRNA antisense | 5’-PUUAACAUCGAUCGUGAUAGUU -3’ | |
| 2^nd^ siRNA sense | 5’- GCAUAUUAAGGUAGAUCCUUU -3’ | |
| 2^nd^ siRNA antisense | 5’- PAGGAUCUACCUUAAUAUGCUU -3’ | |
| 3^rd^ siRNA sense | 5’- ACACAGAUCUGAUGGAUUUUU -3’ | |
| 3^rd^ siRNA antisense | 5’- PAAAUCCAUCAGAUCUGUGUUU -3’ | |
| 4^th^ siRNA sense | 5’- GGGUAGGAUUGACAGGAUUUU -3’ | |
| 4^th^ siRNA antisense | 5’- PAAUCCUGUCAAUCCUACCCUU -3’ | |

Table S2. Primer sequences for PCR

| Primer | | Sequence |
| --- | --- | --- |
| Nppa (ANP) sense | 5’- GATTTCAAGAACCTGCTAGACCAC-3’ | |
| Nppa (ANP) antisense | 5’- CTTCATCGGTCTGCTCGCTC-3’ | |
| FOS (c-fos) sense | 5’- TTTCAACGCGGACTACGAGG -3’ | |
| FOS (c-fos) antisense | 5’- TCGGCTGGGGAATGGTAGTA -3’ | |
| FABP4 sense | 5’- ATGAAAGAAGTGGGAGTTGGC -3’ | |
| FABP4 antisense | 5’- TTCCACGCCCAGTTTGAAGG -3’ | |
| β-actin sense | 5’- CTCTGTGTGGATTGGTGGCT-3’ | |
| β-actin antisense | 5’- CGCAGCTCAGTAACAGTCCG-3’ | |
